# The Biguanides Metformin and Buformin in Combination with 2-Deoxy-glucose or WZB-117 Inhibit the Viability of Highly Resistant Human Lung Cancer Cells

**DOI:** 10.1155/2019/6254269

**Published:** 2019-02-21

**Authors:** Juan Sebastian Yakisich, Neelam Azad, Vivek Kaushik, Anand K. V. Iyer

**Affiliations:** School of Pharmacy, Department of Pharmaceutical Sciences, Hampton University, VA 23668, USA

## Abstract

The biguanides metformin (MET) and to a lesser extent buformin (BUF) have recently been shown to exert anticancer effects. In particular, MET targets cancer stem cells (CSCs) in a variety of cancer types but these compounds have not been extensively tested for combination therapy. In this study, we investigated *in vitro* the anticancer activity of MET and BUF alone or in combination with 2-deoxy-D-glucose (2-DG) and WZB-117 (WZB), which are a glycolysis and a GLUT-1 inhibitor, respectively, in H460 human lung cancer cells growing under three different culture conditions with varying degrees of stemness: (1) routine culture conditions (RCCs), (2) floating lung tumorspheres (LTSs) that are enriched for stem-like cancer cells, and (3) adherent cells under prolonged periods (8-12 days) of serum starvation (PPSS). These cells are highly resistant to conventional anticancer drugs such as paclitaxel, hydroxyurea, and colchicine and display an increased level of stemness markers. As single agents, MET, BUF, 2-DG, and WZB-117 potently inhibited the viability of cells growing under RCCs. Both MET and BUF showed a strong synergistic effect when used in combination with 2-DG. A weak potentiation was observed when used with WZB-117. Under RCCs, H460 cells were more sensitive to MET and BUF and WZB-117 compared to nontumorigenic Beas-2B cells. While LTSs were less sensitive to each single drug, both MET and BUF in combination with 2-DG showed a strong synergistic effect and reduced cell viability to similar levels compared to the parental H460 cells. Adherent cells growing under PPSS were also less sensitive to each single drug, and MET and BUF showed a strong synergistic effect on cell viability in combination with 2-DG. Overall, our data demonstrates that the combination of BGs with either 2-DG or WZB-117 has “broad-spectrum” anticancer activities targeting cells growing under a variety of cell culture conditions with varying degrees of stemness. These properties may be useful to overcome the chemoresistance due to intratumoral heterogeneity found in lung cancer.

## 1. Introduction

The biguanides (BGs) metformin (MET) and to a lesser extent buformin (BUF) have been shown to exert anticancer effects. In particular MET alone or in combination with other anticancer drugs targets cancer stem cells (CSCs) and cancer stem-like cells (CS-LCs) in a variety of cancer types (reviewed by [1]) including lung [2], breast [3], bladder [4], pancreatic cancer [5], and gliomas [6]. At the molecular level, several mechanisms of action linked to multiple pathways critical to tumor growth have been proposed for MET anticancer effects and have been broadly classified into indirect or insulin-dependent pathways and direct or insulin-independent pathways (reviewed by [7]). BGs are also inhibitors of mitochondrial oxidative phosphorylation [8]. Due to its toxicity, it is unlikely that MET at mM concentrations (1-10 mM) can be used in patients since its therapeutic level is about 0.5 ± 0.4 mg/l [9] and plasma levels > 4-10 mg/l (~0.032-0.078 mM) have been associated with lactic acidosis [10, 11]. Indeed, there is a growing consensus that MET alone as monotherapy is unlikely to offer significant clinical benefit but clinical trials with MET in combination therapy with other agents and modalities showed that MET has a broad anticancer activity across a spectrum of malignancies [7]. However, low MET concentrations (0.03–0.3 mM) have been found to inhibit selectively CD44(+)CD117(+) ovarian CSCs through inhibition of EMT and potentiate the effect of cisplatin [12]. BUF has not been extensively tested for combination therapy and at present the effect of this compound on CSCs/CS-LCs has been only evaluated in breast cancer where it was found to inhibit the stemness of breast cancer cells *in vitro* and *in vivo* [13].

Intratumoral heterogeneity, including metabolic heterogeneity, is another factor in general associated with failure of anticancer drugs and of special importance for metabolic inhibitors [14–17]. To be effective, chemotherapeutic regimes should be able to eliminate not only CSCs/CS-LCs but also the bulk populations of non-CSCs/CS-LCs and therefore, intratumoral heterogeneity should be taken in consideration during preclinical drug screening.

The aim of this study is to evaluate *in vitro* the anticancer activity of MET and BUF alone or in combination with 2-deoxy-D-glucose (2-DG) or WZB-117 (WZB) in H460 human lung cancer cells growing under three different culture conditions with varying degrees of chemosensitivity, proliferation, and stemness: (1) routine culture conditions (RCCs), (2) floating lung tumorspheres (LTSs) [18, 19], and (3) adherent cells under prolonged periods (8-12 days) of serum starvation (PPSS) [20]. LTSs (anchorage-independent conditions) and cells growing under PPSS (anchorage-dependent conditions) show increased stemness properties and are highly resistant to conventional anticancer drugs such as paclitaxel, hydroxyurea, colchicine, wortmannin, and LY294002. This strategy partially mimics the intratumoral heterogeneity found in tumors in terms of stemness, proliferation rate, and chemoresistance. 2-Deoxy-D-glucose (2-DG) is a relatively specific inhibitor of glycolysis by binding to the enzyme hexokinase that triggers glucose deprivation without altering other nutrients or metabolic pathways [21]. WZB inhibits the uptake of glucose by inhibiting the activity of the GLUT-1 transporter [22].

## 2. Materials and Methods

### 2.1. Drugs

Metformin (MET), 2-deoxy-D-glucose (2-DG), and necrostatin 1 (Nec1) were purchased from VWR. Buformin hydrochloride (BUF) was purchased from Santa Cruz Biotechnology (Dallas, TX). z-VAD-FMK (zVAD), chloroquine (CQ), and MTT (thiazolyl blue tetrazolium bromide) were purchased from Sigma-Aldrich (St. Louis, MT). The stock solution of MET and 2-DG and BUF (100 mM) was prepared in distilled sterile water and stored in aliquots at -20°C. WZB-117 was purchased from Sigma-Aldrich (St. Louis, MO), prepared as a stock solution (10 mM) in DMSO and stored in aliquots at -20°C. Stock solutions of Nec1 (10 mM) and zVAD (10 mM) were done in DMSO and stored in aliquots at -20°C. CQ was prepared as a stock solution (10 mM) in distilled sterile water, filter sterilized, and stored in aliquots at -20°C.

### 2.2. Cell Culture

The human lung epithelial cancer cell line NCI-H460 (ATCC Cat# HTB-177, RRID:CVCL_0459) and the normal human bronchial epithelial Beas-2B cell line (ATCC Cat# CRL-9609, RRID:CVCL_0168) were obtained from the American Type Culture Collection (Manassas, VA). H460 cells are considered highly resistant to chemotherapy [23]. To standardize culture conditions, all cell lines were cultured in RPMI 1640 supplemented with 5% FBS, 2 mM L-glutamine, 100 U/ml penicillin, and 100 mg/ml streptomycin. As previously reported, NCI-H460 [24] and Beas-2B cells [25] are able to grow well in a RPMI-1640 medium. All cells were cultured in a 5% CO_2_ environment at 37°C.

### 2.3. Short-Term Viability Assay for Adherent Cells

Cells (~2,000 cells per well) were plated in 96-well cell-culture microplates (Costar, USA) and incubated overnight in a cell culture medium to allow them to adhere. Cells were then exposed to the appropriate concentration of drug or vehicle for 72 hours. Cell viability was evaluated by the MTT assay. The absorbance of solubilized formazan was read at 570 nm using an ELISA reader (Bio-TEK, Synergy-1). In all cases, the highest concentration of DMSO was used in the control and this concentration was maintained at ~0.25% (*v*/*v*). This DMSO concentration did not show any significant antiproliferative effect on the cell line in a short-term assay.

### 2.4. Colony-Forming Assay

Colony-forming assay was performed as previously described [26, 27]. Briefly, 200 cells/well were plated in 6-well plates and allowed to adhere overnight. Cells were then treated with drugs at the indicated concentration or with vehicle alone for 72 h in complete media (CM: DMEM containing 5% FBS). After drug exposure, cells were incubated with complete media for 9 days (media were changed every 72 h). Then, cells were fixed with 3.7% formaldehyde for 60 min, stained with 0.01% crystal violet, and photographed. Colonies were counted using ImageJ software (ImageJ 1.49 v, http://imagej.nih.gov/ij/).

### 2.5. Generation of Lung Tumorspheres (LTs) and Determination of Tumorsphere Viability

A detailed protocol for the generation of floating lung tumorspheres (LTs) grown in the absence of any external mitogenic stimulation and the determination of tumorsphere viability by the CCK assay can be found in [18]. Briefly, H460 cells grown in CM (70-80% confluency) were cultured overnight in serum-free media (SFM: same as CM but without FBS). Then, cells were trypsinized and incubated in SFM for at least 14 days in poly-HEMA-coated plates to prevent attachment. For maintenance of LTs, the SFM was replaced every 3-4 days. LTs grown in SFM for 14-21 days were used for subsequent experiments. The viability of floating LT cells growing in poly-HEMA plates was measured by the CCK assay (Dojindo Laboratories) as follows: FTs were collected in 15 ml Falcon tubes, centrifuged at 700 rpm × 3 min and resuspended in fresh SFM. In order to plate the same number of cells, this cell suspension was split into 1 ml aliquots. Vehicle or drugs were added to each aliquot, and then, 150 *μ*l cell suspension was loaded into each microwell (in a 96-well plate) and incubated for 72 h. After incubation, 15 *μ*l of the WST-8 solution was added to each microwell, incubated for 60-120 min and the absorbance was read at 450 nm using an ELISA reader (Bio-TEK, Synergy-1).

### 2.6. Western Blotting

Preparation of cell lysates and Western blotting was performed as described previously [28]. Antibodies for TFAM (transcription factor A, mitochondrial; aka TCF6; Cell Signaling Technology Cat# 8076S RRID:AB_10949110), SOD2 (manganese superoxide dismutase (MnSOD, Cell Signaling Technology Cat# 13141, RRID:AB_2636921)), CDK2 (Cell Signaling Technology Cat# 2546S; RRID:AB_2276129), cyclin E (Cell Signaling Technology Cat # 20808S), AMPK*α* (Cell Signaling Technology Cat# 5831S, RRID:AB_10622186), AMPK*α* (Thr172 (Cell Signaling Technology Cat# 2535S, RRID:AB_331250)), and GADPH (Santa Cruz Biotechnology Cat# sc-25778, RRID:AB_10167668) were purchased from Santa Cruz Biotechnology (Santa Cruz, CA). Peroxidase-conjugated secondary antibody (Cell Signaling Technology Cat# 7074 RRID:AB_2099233) was purchased from Cell Signaling (Danvers, MA, USA). The immune complexes were detected by chemiluminescence and quantified using analyst/PC densitometry software (Bio-Rad Laboratories, Hercules, CA).

### 2.7. Flow Cytometry

Flow cytometry experiments were done as previously described [29]. Briefly, cells grown in 100 mm Petri dishes were treated with drugs or vehicle for 24 hours and collected by trypsinization, washed twice with PBS, and fixed overnight in 70% ethanol at 4°C. After two washes with PBS, cells were treated with DNAse-free RNAse (100 *μ*g/ml) and stained with propidium iodide (50 *μ*g/ml). Cell cycle analysis was performed using the ACEA NovoCyte 2060 instrument (ACEA Biosciences, San Diego, CA) and NovoExpress (version 1.0.2) software. The cell cycle distribution is shown as the percentage of cells containing G_0_/G_1_, S, and G_2_/M DNA as identified by propidium iodide staining.

### 2.8. Statistical Analysis

**Figure 1:**
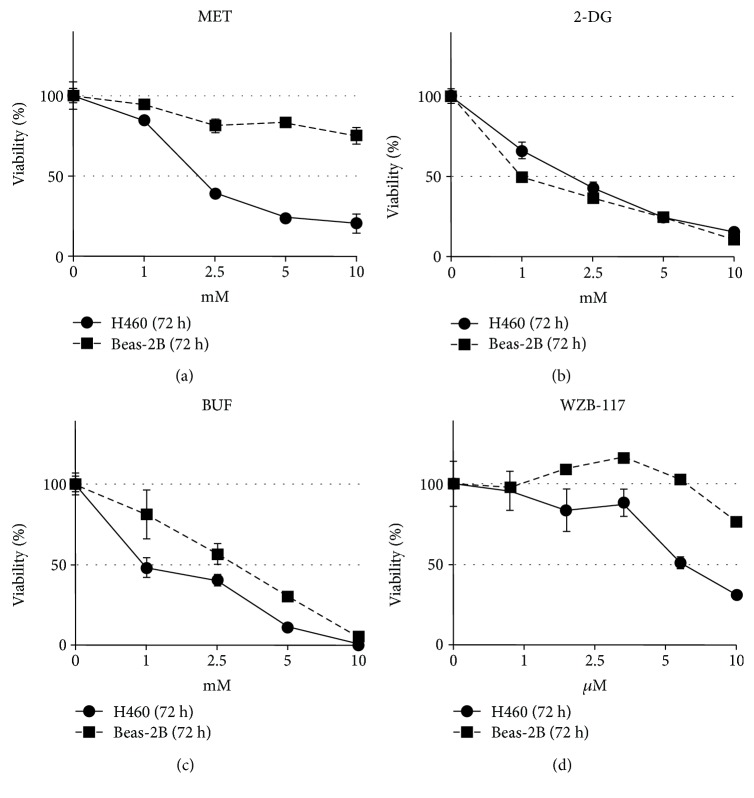
Metformin (MET) and buformin (BUF) preferentially inhibit viability of human H460 cancer cells compared to human noncancer Beas-2B cells. Cells growing under RCCs were incubated with the indicated concentrations of drugs for 72 h. Control cells (DMSO) were incubated with equivalent concentration of DMSO. The viability was measured by the MTT assay.

**Figure 2:**
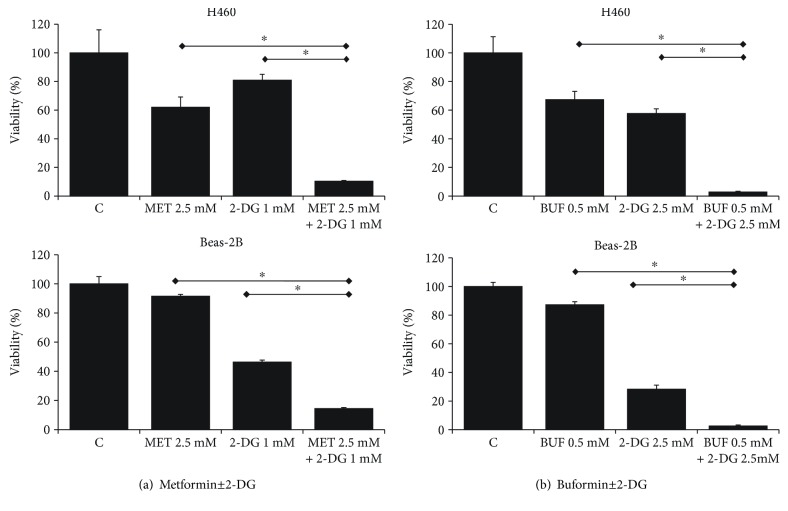
Metformin (MET) and buformin (BUF) in combination with 2-DG have a synergistic effect on cell viability of H460 lung cancer cells. Cells growing under RCCs were incubated with the indicated concentrations of drugs for 72 h. Control cells (DMSO) were incubated with equivalent concentration of DMSO. The viability was measured by the MTT assay. ^∗^ indicates *P* < 0.001 and *P* < 0.05, respectively (ANOVA).

**Figure 3:**
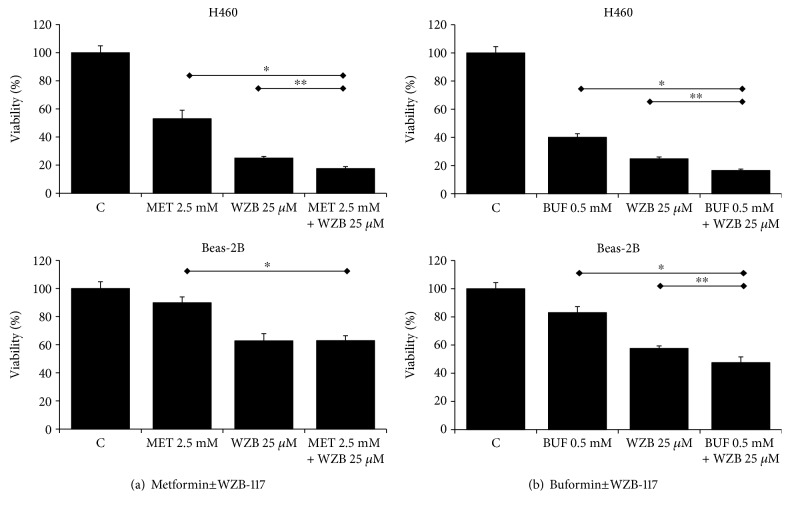
Metformin and buformin in combination with WZB-117 have a synergistic effect on cell viability of H460 lung cancer cells. Cells growing under RCCs were incubated with the indicated concentrations of drugs for 72 h. Control cells (DMSO) were incubated with equivalent concentration of DMSO. The viability was measured by the MTT assay. ∗ and ∗∗ indicate *P* < 0.001 and *P* < 0.05, respectively (ANOVA).

**Figure 4:**
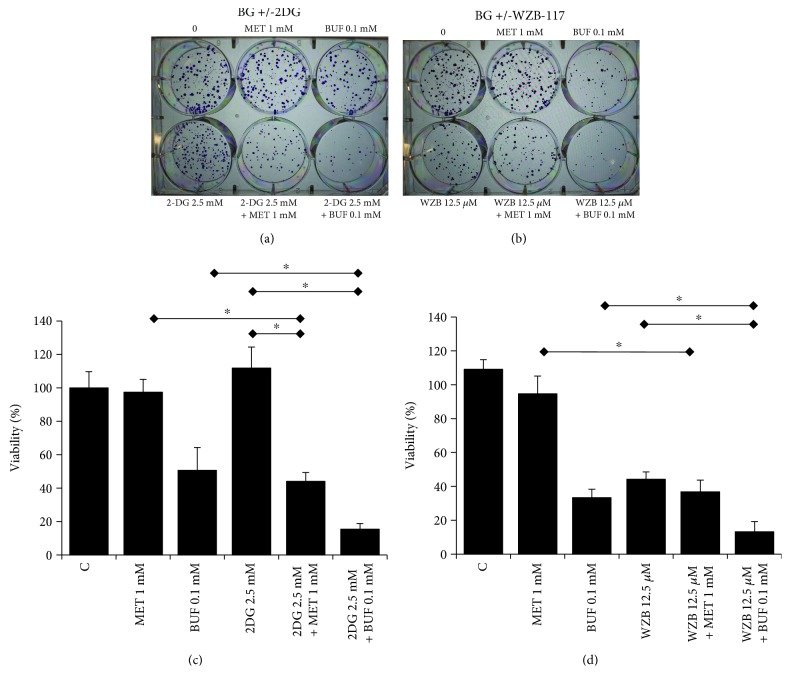
Metformin and buformin alone or in combination with 2-DG decrease clonogenicity of H460 cells. Cells were incubated with the indicated concentration of drugs for 72 hours followed by incubation in drug-free media for ~9 days. Data (mean ± SD) is representative of two independent experiments performed in triplicates.

**Figure 5:**
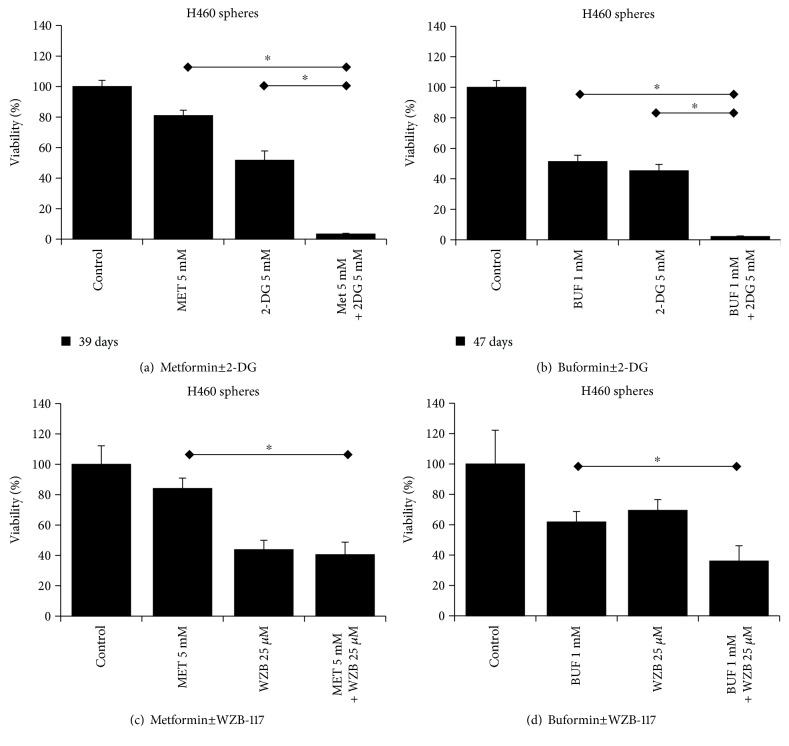
Metformin and buformin in combination with 2-DG or WZB-117 inhibit the viability of H460 lung cancer cells growing as floating tumorspheres (anchorage-independent). The bars indicated the mean OD of sphere-forming H460 cells after treatments with different concentrations of drugs measured by the CCK assay. ∗ indicates *P* < 0.001 and *P* < 0.05, respectively (ANOVA).

**Figure 6:**
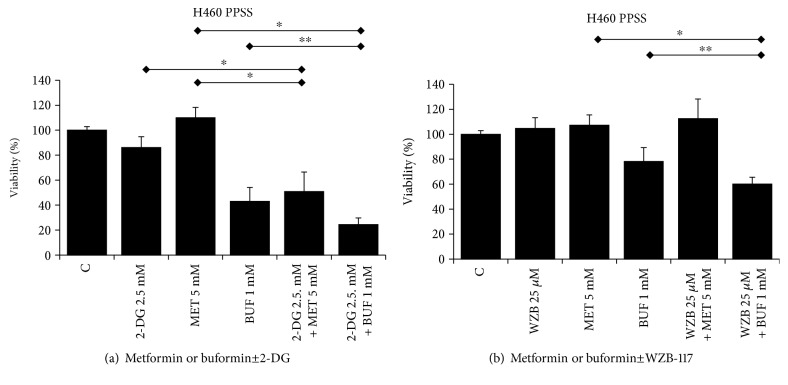
Metformin and buformin in combination with 2-DG or WZB-117 inhibit the viability of H460 lung cancer cells growing under PPSS (anchorage-dependent). Cells growing under PPSS were incubated with the indicated concentrations of drugs for 72 h. Control cells (DMSO), were incubated with equivalent concentration of DMSO. The viability was measured by the MTT assay. ∗ and ∗∗ indicate *P* < 0.001 and *P* < 0.05, respectively (ANOVA).

**Figure 7:**
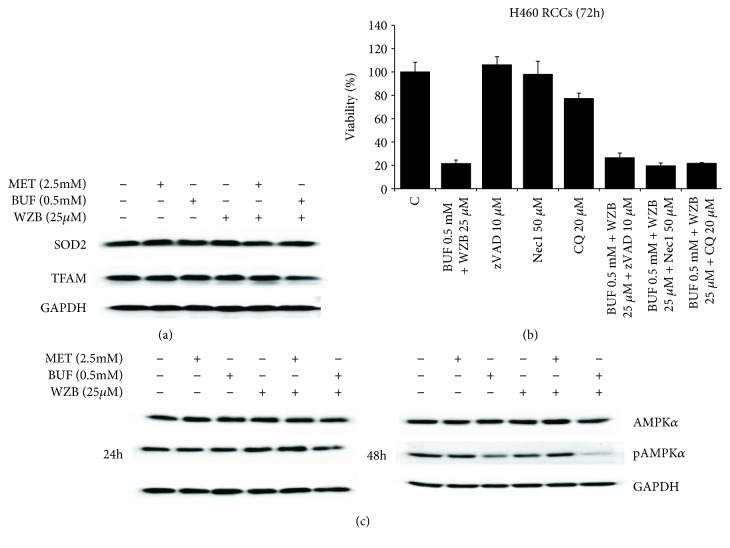
Low concentrations of metformin (MET) and buformin (BUF) alone or in combination with WZB-117 (WZB) do not impair mitochondrial function nor induce cell death. (a) Cells were treated with the indicated concentrations of drugs for 48 h, and the expression of TFAM and SOD2 was analyzed by Western blotting. GADPH was used as loading control. (b) Apoptosis, necroptosis, or autophagy inhibitors did not prevent the inhibitory effect of BUF+WZB on the viability in H460 cells. Cells growing under RCCs were incubated with BUF (0.5 mM)+WZB (25 *μ*M) alone or in the presence of zVAD, Nec1, or CQ for 72 h. Cell viability was measured by the MTT assay. Results (*X* ± SD) are representative of two independent experiments performed in sextuplicates. (c) Low concentrations of BUF alone or in combination with WZB decreased the expression of pAMPK*α*. Cells were treated with the indicated concentrations of drugs for 24 or 48 h, and the expression of AMPK*α* and pAMPK*α* was analyzed by Western blotting. GADPH was used as loading control.

The IC_50_ (drug concentration inhibiting cell growth by 50%) was determined by interpolation from the dose-response curves using a sigmoidal logistic 3 parameter equation. Each point represents the mean ± standard deviation (SD) of sextuplicate wells (see Figures 1–7 for details). Curve fitting and all pairwise multiple comparison procedures (analysis of variance (ANOVA) and Student–Newman–Keuls method) and Student's *t*-test have been done using SigmaPlot (version 11.0) software.

## 3. Results

### 3.1. Biguanides in Combination with 2-Deoxy-D-glucose Has Synergistic Effect on the Viability of Lung Cancer Cells

We first characterized the inhibitory effect of MET, BUF, 2-DG, and WZB-117 as single agents on the viability of Beas-2B and H460 cells growing as monolayers under routine culture conditions. While MET, BUF, and WZB-117 were more effective against H460 cancer cells compared to Beas-2B cells, 2-DG showed similar potency against both Beas-2B and H460 cells (Figure 1). We next evaluated the effect of MET or BUF in combination with 2-DG or WZB-117. Both MET and BUF in combination with 2-DG showed a synergistic effect and showed similar potency toward H460 and to Beas-2B cells (Figure 2). When MET and BUF were used in combination with WZB-117, the synergistic effect was minimum but more efficient toward H460 cells compared to Beas-2B (Figure 3).

### 3.2. Biguanides Alone or in Combination with 2-DG or WZB-117 Inhibit the Clonogenicity of H460 Cells

The effect of BGs alone or in combination with 2DG or WZB on the ability of H460 cells to form colonies was evaluated by the colony-forming assay. As single agents, both MET and BUF were able to reduce the number of colonies compared to controls. The concentration required to decrease the number of colonies was much lower compared to the IC_50_ measured by viability assays. For instance, while the IC_50_ for BUF using the MTT assay was ~1 mM, only 0.1 mM was able to reduce the number of colonies to approximately 10-20%. In agreement with the viability assays (Figures 2 and 3) when MET or BUF were used in combination with either 2-DG or WZB-117, a synergistic effect was observed (Figure 4).

### 3.3. Biguanides in Combination with 2-Deoxy-D-glucose Has Synergistic Effect on the Viability of Lung Cells with Increased Chemoresistance

The effects of BGs alone or in combination with 2DG or WZB on cells with increased stemness were evaluated in LTs that are enriched for CSCs/CS-LCs. As single agents, both BGs and 2DG and WZB significantly inhibited the viability of LTs. However, BUF showed a more potent effect compared to MET. MET or BUF in combination with 2DG showed a strong potentiation effect on cell viability of LTs. This effect was minor when MET or BUF was used in combination with WZB (Figure 5). We also evaluated the ability of these drugs on cells growing under PPSS that showed a similar trend (Figure 6) confirming that BGs in combination with 2DG can effectively target cells with increased chemoresistance.

### 3.4. Biguanides and WZB-117 Do Not Induce Mitochondrial Dysfunction or Cell Death but Induce Cell Cycle Arrest

**Figure 8:**
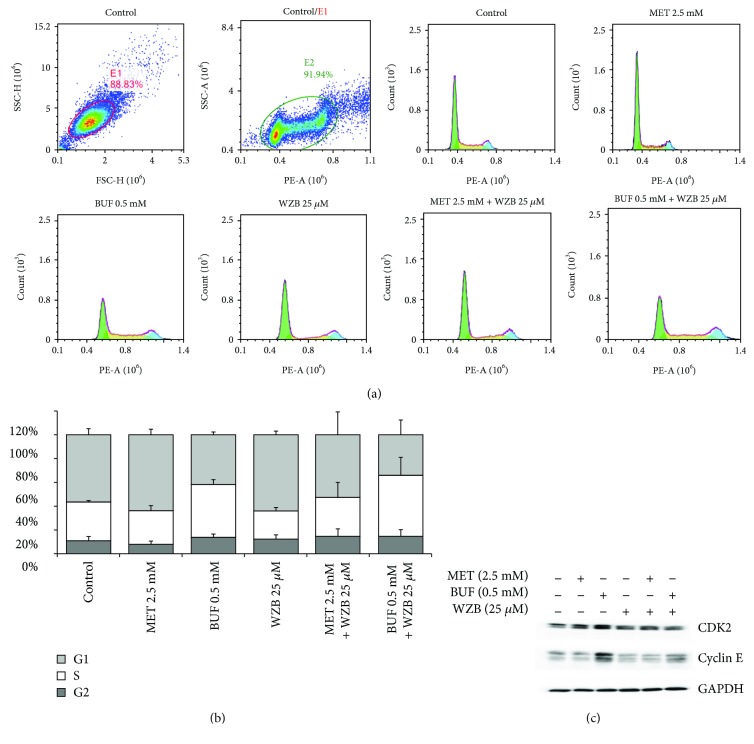
Effect of low concentrations of metformin (MET) and buformin (BUF) alone or in combination with WZB-117 (WZB) on cell cycle distribution. H460 cells were treated with the indicated concentration of drugs for 24 h and then evaluated by flow cytometry as described in Materials and Methods (a-b). (c) Expression of S-phase markers (CDK2 and cyclin E) in H460 cells treated for 24 h with the indicated concentration of drugs.

In order to investigate the mechanism involved in the effect of BGs and WZB-117 on cell viability, cells grown in 100 mm Petri dishes were incubated with MET (2.5 mM), BUF (0.5 mM), WZB (25 *μ*M), MET (2.5 mM)+WZB (25 *μ*M), or BUF (0.5 mM)+WZB(25 *μ*M) for 24 h and protein lysates were collected for Western blot analysis. Control cells were treated with equivalent concentrations of vehicle (DMSO+H_2_O).  Figure 7(a) shows that each drug alone or in combination did not significantly alter the expression of mitochondrial function markers (SOD and TFAM) or key apoptosis or autophagy markers (data not shown). However, BUF alone or in combination with WZB decreased the expression of pAMPK*α*, a key regulator of fatty acid and glucose metabolism. This effect was clearly observed after 48 h treatment (Figure 7(c)). In addition, pharmacological inhibition of apoptosis, autophagy, or necroptosis with zVAD, CQ, or Nec1 at concentrations that effectively inhibit these pathways in H460 cells [30] did not have any effect on the inhibitory effect of WZB±BGs. Cell cycle analysis by flow cytometry showed that exposure to WZB+BUF for 48 h increased the percentage of cells in the S phase (Figures 8(a) and 8(b)). This result was supported by Western blot analysis that showed increased expression of the S-phase markers CDK2 and cyclin E (Figure 8(c)).

## 4. Discussion

The effects of BGs such as MET and to a lesser extent BUF in combination with 2-DG have been evaluated in few cancer types [31–34]. At present, there is no information in lung cancers and there are no studies of the use of these BGs in combination with WZB. In this study, we investigated the effects of MET and BUF alone or in combination with 2-DG or WZB-117 in the H460 lung cancer cell line and in the Beas-2B noncancerous lung cell line.

Consistent with others, we found that MET (IC_50_~2.9 mM) or BUF (IC_50_~1 mM) alone at mM concentrations has an inhibitory effect on the viability of H460 cells. H460 cancer cells seem to be less sensitive to BUF alone compared to other cancers. For comparison, Kilgore et al. recently reported that MET and BUF inhibited the viability of two endometrial cancer cells (ECC-1 and Ishikawa cell lines) with IC_50_ of 1.6 and 1.4 mM for MET and IC_50_ of 0.15 and 0.08 mM for BUF. The BUF concentration necessary to inhibit the viability of H460 cells was also in the mM range (IC_50_~1 mM).

Both BGs showed less toxicity toward noncancer Beas-2B cells. In Beas-2B cells, the IC_50_ for MET and BUF were >10 mM and ~2.5 mM, respectively (Figure 1). While 2-DG was found to have a similar effect on cancer (H460) and noncancer cells (Beas-2B), WZB showed stronger effects on Beas-2B cells. MET in combination with 2-DG showed similar toxicity to both cell types. This is likely because Beas-2B cells were slightly more sensitive to 2-DG compared to H460 cells.

BUF inhibited the clonogenicity of H460 cells at a very low concentration: only 0.1 mM was required to inhibit the number of colonies by approximately 50%. This result indicates that in addition to its ability to decrease cell viability, BUF is even more potent in inhibiting the ability of H460 cells to produce progeny. This effect was also potentiated when used in combination with 2-DG or WZB (Figure 4). We also analyzed the effect of MET or BUF alone or in combination with 2-DG or WZB in two models of multidrug-resistant cells: (i) cells growing under PPSS (anchorage-dependent) and (ii) lung tumorspheres (anchorage-independent) that are enriched for CSCs/CS-LCs. At present, there are few reports on the effect of BUF alone on cancer cells and there is no information on the effect of BUF on lung CSCs/CS-LCs. We are the first to report a strong effect of BUF alone on the cell viability of lung tumorspheres. In both multidrug-resistant experimental models, MET and BUF in combination with 2-DG or WZB significantly decreased cell viability. At the molecular level, we examined the effect of MET and BUF in combination with WZB. Since BGs are known inhibitors of mitochondrial oxidative phosphorylation and they have been shown to increase reactive oxygen species (ROS), we analyzed mitochondrial function by assessing the expression of TFAM and SOD2. TFAM plays an essential role in ATP production by maintaining mtDNA integrity [35]. SOD2 is a mitochondrial detoxification enzyme that catalyzes the conversion of superoxide to hydrogen peroxide and a key component antioxidant defense from ROS [36]. Neither MET nor BUF alone or in combination significantly altered the expression of TFAM or SOD2 (Figure 7) indicating that these drugs do not significantly impair mitochondrial function. On the other hand, none of the treatments altered the expression of key apoptotic markers such as PARP or caspase 9 (data not shown). We observed that BUF alone or in combination with WZB induced an important downregulation of the expression of phosphorylated AMPK*α* (p-AMPK*α* Thr172). Such effect was not observed in cells treated with MET 2.5 mM (Figure 7(c)). Guo et al. showed that in H460 cells, MET, only at concentrations higher than 4-6 mM, significantly increased the expression of pAMPK [37] that is in agreement with our observation. It is also important to notice that the effect of MET on pAMPK*α* may be time dependent since it was reported in the AGS gastric cancer cell line that MET (10 mM) induced a transient increase of pAMPK*α* peaking at 8 h and returning to basal levels after 24 h [38]. Flow cytometry and Western blot analysis of the S-phase protein markers CDK2 and cyclin E (Figure 8) demonstrated that WZB+BUF arrested cells at the S-phase. Regarding MET, we observed a small increase of the G_1_ fraction at the concentration tested (2.5 mM). This result is in agreement with Guo et al. who reported that 5 and 10 mM induced a G_0_-G_1_ arrest of approximately 5% and 15%, respectively [37]. Microscopic observation showed extensive cell death after prolonged exposure (>7 days) to MET, BUF, MET+WZB, or BUF+WZB (data not shown). These results suggest that these drugs at relatively low concentration exert an early cytostatic effect followed by late activation of cell death by mechanisms yet to be identified.

## 5. Conclusions

In this study, we demonstrated that the BGs MET and BUF inhibited the viability of human H460 lung cancer with relatively more potency when compared to the human noncancer lung epithelial cell line Beas-2B. BGs in combination with either 2-DG or WZB-117 showed “broad-spectrum” anticancer activities targeting cells with varying degrees of chemoresistance. These results warrant further studies to evaluate their potential to overcome the inherent chemoresistance of lung cancer due to intratumoral heterogeneity.

## Data Availability

The data used to support the findings of this study are included within the article.

## References

[1] Zhang H. H., Guo X. L. (2016). Combinational strategies of metformin and chemotherapy in cancers. *Cancer Chemotherapy and Pharmacology*.

[2] Xiao Z., Sperl B., Ullrich A., Knyazev P. (2014). Metformin and salinomycin as the best combination for the eradication of NSCLC monolayer cells and their alveospheres (cancer stem cells) irrespective of EGFR, KRAS, EML4/ALK and LKB1 status. *Oncotarget*.

[3] Lee K. M., Lee M., Lee J. (2016). Enhanced anti-tumor activity and cytotoxic effect on cancer stem cell population of metformin-butyrate compared with metformin HCl in breast cancer. *Oncotarget*.

[4] Liu Q., Yuan W., Tong D. (2016). Metformin represses bladder cancer progression by inhibiting stem cell repopulation via COX2/PGE2/STAT3 axis. *Oncotarget*.

[5] Ning X., du Y., Ben Q. (2016). Bulk pancreatic cancer cells can convert into cancer stem cells(CSCs) in vitro and 2 compounds can target these CSCs. *Cell Cycle*.

[6] Yu Z., Zhao G., Li P. (2016). Temozolomide in combination with metformin act synergistically to inhibit proliferation and expansion of glioma stem-like cells. *Oncology Letters*.

[7] Gong J., Kelekar G., Shen J., Shen J., Kaur S., Mita M. (2016). The expanding role of metformin in cancer: an update on antitumor mechanisms and clinical development. *Targeted Oncology*.

[8] Bridges H. R., Jones A. J. Y., Pollak M. N., Hirst J. (2014). Effects of metformin and other biguanides on oxidative phosphorylation in mitochondria. *The Biochemical Journal*.

[9] Lalau J. D., Lemaire-Hurtel A. S., Lacroix C. (2011). Establishment of a database of metformin plasma concentrations and erythrocyte levels in normal and emergency situations. *Clinical Drug Investigation*.

[10] Lalau J. D., Kajbaf F. (2014). Interpreting the consequences of metformin accumulation in an emergency context: impact of the time frame on the blood metformin levels. *International Journal of Endocrinology*.

[11] Vecchio S., Giampreti A., Petrolini V. M. (2014). Metformin accumulation: lactic acidosis and high plasmatic metformin levels in a retrospective case series of 66 patients on chronic therapy. *Clinical Toxicology*.

[12] Zhang R., Zhang P., Wang H. (2015). Inhibitory effects of metformin at low concentration on epithelial-mesenchymal transition of CD44^+^CD117^+^ ovarian cancer stem cells. *Stem Cell Research & Therapy*.

[13] Parris A. B., Zhao Q., Howard E. W., Zhao M., Ma Z., Yang X. (2017). Buformin inhibits the stemness of erbB-2-overexpressing breast cancer cells and premalignant mammary tissues of MMTV-erbB-2 transgenic mice. *Journal of Experimental & Clinical Cancer Research*.

[14] Rustum Y. M., Tóth K., Seshadri M. (2010). Architectural heterogeneity in tumors caused by differentiation alters intratumoral drug distribution and affects therapeutic synergy of antiangiogenic organoselenium compound. *Journal of Oncology*.

[15] Neelakantan D., Drasin D. J., Ford H. L. (2014). Intratumoral heterogeneity: clonal cooperation in epithelial-to-mesenchymal transition and metastasis. *Cell Adhesion & Migration*.

[16] Mansuet-Lupo A., Zouiti F., Alifano M. (2014). Intratumoral distribution of EGFR mutations and copy number in metastatic lung cancer, what impact on the initial molecular diagnosis?. *Journal of Translational Medicine*.

[17] Kang S. R., Song H. C., Byun B. H. (2014). Intratumoral metabolic heterogeneity for prediction of disease progression after concurrent chemoradiotherapy in patients with inoperable stage III non-small-cell lung cancer. *Nuclear Medicine and Molecular Imaging*.

[18] Yakisich J. S., Azad N., Kaushik V., Iyer A. K. V. (2017). Cancer cell plasticity: rapid reversal of chemosensitivity and expression of stemness markers in lung and breast cancer tumorspheres. *Journal of Cellular Physiology*.

[19] Yakisich J. S., Azad N., Venkatadri R. (2016). Formation of tumorspheres with increased stemness without external mitogens in a lung cancer model. *Stem Cells International*.

[20] Yakisich J. S., Venkatadri R., Azad N., Iyer A. K. V. (2017). Chemoresistance of lung and breast cancer cells growing under prolonged periods of serum starvation. *Journal of Cellular Physiology*.

[21] Wang Q., Liang B., Shirwany N. A., Zou M. H. (2011). 2-Deoxy-D-glucose treatment of endothelial cells induces autophagy by reactive oxygen species-mediated activation of the AMP-activated protein kinase. *PLoS One*.

[22] Liu Y., Cao Y., Zhang W. (2012). A small-molecule inhibitor of glucose transporter 1 downregulates glycolysis, induces cell-cycle arrest, and inhibits cancer cell growth *in vitro* and *in vivo*. *Molecular Cancer Therapeutics*.

[23] Coughlin S. S., Matthews-Juarez P., Juarez P. D., Melton C. E., King M. (2014). Opportunities to address lung cancer disparities among African Americans. *Cancer Medicine*.

[24] Medan D., Luanpitpong S., Azad N. (2012). Multifunctional role of Bcl-2 in malignant transformation and tumorigenesis of Cr(VI)-transformed lung cells. *PLoS One*.

[25] Dong P., Fu X., Wang X., Wang W. M., Cao W. M., Zhang W. Y. (2015). Protective effects of sesaminol on BEAS-2B cells impaired by cigarette smoke extract. *Cell Biochemistry and Biophysics*.

[26] Kaushik V., Azad N., Yakisich J. S., Iyer A. K. V. (2017). Antitumor effects of naturally occurring cardiac glycosides convallatoxin and peruvoside on human ER+ and triple-negative breast cancers. *Cell Death Discovery*.

[27] Rafehi H., Orlowski C., Georgiadis G. T., Ververis K., el-Osta A., Karagiannis T. C. (2011). Clonogenic assay: adherent cells. *Journal of Visualized Experiments*.

[28] Kaushik V., Yakisich J. S., Azad N. (2017). Anti-tumor effects of cardiac glycosides on human lung cancer cells and lung tumorspheres. *Journal of Cellular Physiology*.

[29] Yakisich J. S., Azad N., Kaushik V., O'Doherty G. A., Iyer A. K. (2017). Nigericin decreases the viability of multidrug-resistant cancer cells and lung tumorspheres and potentiates the effects of cardiac glycosides. *Tumour Biology*.

[30] Yakisich J. S., Kulkarni Y., Azad N., Iyer A. K. V. (2017). Selective and irreversible induction of necroptotic cell death in lung tumorspheres by short-term exposure to verapamil in combination with sorafenib. *Stem Cells International*.

[31] Chatterjee S., Thaker N., De A. (2015). Combined 2-deoxy glucose and metformin improves therapeutic efficacy of sodium-iodide symporter-mediated targeted radioiodine therapy in breast cancer cells. *Breast Cancer*.

[32] Hou X. B., Li T. H., Ren Z. P., Liu Y. (2016). Combination of 2-deoxy d-glucose and metformin for synergistic inhibition of non-small cell lung cancer: a reactive oxygen species and P-p38 mediated mechanism. *Biomedicine & Pharmacotherapy*.

[33] Saito S., Furuno A., Sakurai J. (2009). Chemical genomics identifies the unfolded protein response as a target for selective cancer cell killing during glucose deprivation. *Cancer Research*.

[34] Zhu J., Zheng Y., Zhang H., Sun H. (2016). Targeting cancer cell metabolism: the combination of metformin and 2-deoxyglucose regulates apoptosis in ovarian cancer cells via p38 MAPK/JNK signaling pathway. *American Journal of Translational Research*.

[35] Kang D., Kim S. H., Hamasaki N. (2007). Mitochondrial transcription factor A (TFAM): roles in maintenance of mtDNA and cellular functions. *Mitochondrion*.

[36] Minig V., Kattan Z., van Beeumen J., Brunner E., Becuwe P. (2009). Identification of DDB2 protein as a transcriptional regulator of constitutive SOD2 gene expression in human breast cancer cells. *The Journal of Biological Chemistry*.

[37] Guo Q., Liu Z., Jiang L. (2016). Metformin inhibits growth of human non-small cell lung cancer cells via liver kinase B-1-independent activation of adenosine monophosphate-activated protein kinase. *Molecular Medicine Reports*.

[38] Huang D., He X., Zou J. (2016). Negative regulation of Bmi-1 by AMPK and implication in cancer progression. *Oncotarget*.

